# Endocannabinoid System: Chemical Characteristics and Biological Activity

**DOI:** 10.3390/ph16020148

**Published:** 2023-01-19

**Authors:** Bismarck Rezende, Allan Kardec Nogueira Alencar, Graziele Freitas de Bem, Fabrícia Lima Fontes-Dantas, Guilherme Carneiro Montes

**Affiliations:** 1 Department of Pharmacology and Psychobiology, Roberto Alcântara Gomes Institute Biology (IBRAG), Rio de Janeiro State University (UERJ), Rio de Janeiro 20551-030, Brazil; 2 Department of Biomedical Engineering, Tulane University, New Orleans, LA 70118, USA

**Keywords:** endocannabinoid system, receptor cannabinoid, endocannabinoid ligands, phytocannabinoids, chronic pain, mood disorders, integrative and complementary health practices

## Abstract

The endocannabinoid system (eCB) has been studied to identify the molecular structures present in *Cannabis sativa*. eCB consists of cannabinoid receptors, endogenous ligands, and the associated enzymatic apparatus responsible for maintaining energy homeostasis and cognitive processes. Several physiological effects of cannabinoids are exerted through interactions with various receptors, such as CB1 and CB2 receptors, vanilloid receptors, and the recently discovered G-protein-coupled receptors (GPR55, GPR3, GPR6, GPR12, and GPR19). Anandamide (AEA) and 2-arachidoylglycerol (2-AG), two small lipids derived from arachidonic acid, showed high-affinity binding to both CB1 and CB2 receptors. eCB plays a critical role in chronic pain and mood disorders and has been extensively studied because of its wide therapeutic potential and because it is a promising target for the development of new drugs. Phytocannabinoids and synthetic cannabinoids have shown varied affinities for eCB and are relevant to the treatment of several neurological diseases. This review provides a description of eCB components and discusses how phytocannabinoids and other exogenous compounds may regulate the eCB balance. Furthermore, we show the hypo- or hyperfunctionality of eCB in the body and how eCB is related to chronic pain and mood disorders, even with integrative and complementary health practices (ICHP) harmonizing the eCB.

## 1. Introduction

*Cannabis sativa* has been used for recreational [1,2], therapeutic [3,4], and other purposes for thousands of years [5]. The plant contains more than 120 terpenes called phytocannabinoids, including one of the main and most recognized representatives, Δ9-tetrahydrocannabinol (THC). The molecular structure of Δ9-THC was identified for the first time in 1964, which led to the supposition of the existence of a cannabinoid receptor and boosted the discovery of the endocannabinoid system (eCB), which is largely an intercellular system that is responsible for energy homeostasis and regulates food intake, metabolism, and energy expenditure, maintaining a consistent body weight [6]. In addition to appetite, eCB might contribute to cognitive processes linked to memory, mood, and pain [7]. eCB has gained prominence during the COVID-19 pandemic, not only for the inhibition of SARS-CoV-2 replication but also in different studies that include its use for the treatment of chronic pain and mood disorders [8,9,10,11,12,13]. eCB is an active system that stimulates a complex cell signaling network. It involves a combination of cannabinoid receptors, endogenous cannabinoids (endocannabinoids), and enzymes responsible for the synthesis and degradation of endocannabinoids. The first studies started with the identification of receptors named type 1 and 2 cannabinoid receptors, or CB1R and CB2R [14,15,16,17]. Moreover, there was the discovery of endogenous ligands, which enhanced our knowledge of new compounds, such as *N*-arachidonoylethanolamide, the first endocannabinoid molecule to be discovered, which was named “anandamide”, a Sanskrit word meaning “bliss” or extreme happiness [18], followed by the identification of 2-arachidonoylglycerol (2-AG), which together with the enzymes responsible for the synthesis and degradation of these compounds, make up what we know today as the endocannabinoid system [19].

In October 2022, a PubMed search for scientific journal articles published in all available periods containing the word “Endocannabinoid” revealed 12.272 results, with increased interest in studies and publications since 2000. These numbers illustrate the rapidly increasing financial support in recent years as well as the scientific interest in understanding the molecular mechanisms in different contexts of clinical application. This review focuses on recent advances in the understanding of eCB components and discusses the roles of phytocannabinoids, other exogenous compounds, the treatment of pain and mood disorders using eCB, and integrative and complementary health practices.

## 2. Cannabinoid Receptors

**Figure 1:**
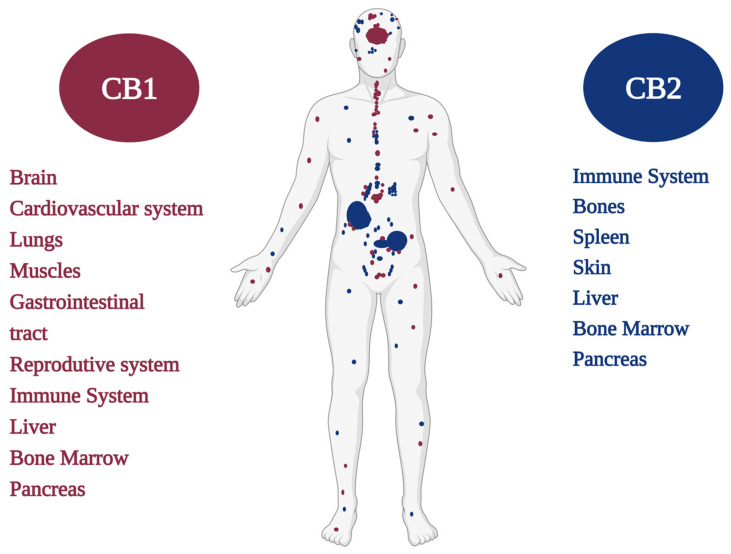
CB1 and CB2 cannabinoid receptors and their distribution in the human body.

CB1/CB2 cannabinoid receptors are mainly distinguished by the sequence of amino acids in the polypeptide chain and by their distributions in different tissues [20,21,22,23] (Figure 1). Pharmacological studies suggest that cannabinoid molecules might act on receptors other than the classic CB1 and CB2 receptors, such as the vanilloid receptors TRPV1, TRPV2, TRPV3, TRPV4, TRPA1, TRPM8 and metabotropic receptors such as GPR55, GPR3, GPR6, GPR12, and GPR19, among other receptors, as well as enzymes and proteins [24,25,26]. Recently, the eCB has been expanded, and researchers have named it the endocannabinoidome (eCBome), a meaningful reference that includes all components as well as proteins, enzymes, and lipids that are directly or indirectly involved in cannabinoid system modulation and significantly affect health [27].

The endogenous lipid signaling system might be deeply involved in several physiological conditions and pathological disorders and may provide a future perspective for the treatment of different illnesses [28,29,30,31].

Phytocannabinoids, such as cannabidiol (CBD), have wide therapeutic applicability, possibly because of their ability to target numerous receptors. The eCBome plays a role in the microbiota–gut–brain axis, which has emerged as an important player in the control of affective and cognitive functions and their pathological changes. However, the molecular and biochemical bases of the interaction and the biological relationships of the new receptor subtypes with cannabinoid ligands have not been fully elucidated; therefore, further studies are needed [32,33,34].

The type 1 cannabinoid receptor (CB1R) is encoded by the CNR1 gene [35,36] and was cloned in rats by Matsuda et al. in 1990 [14]. Years later, CB1R was also cloned in human tissues [37,38] and mice [39], exhibiting 97–99% amino acid sequence identity between these species [40]. After receptor cloning, it was possible to design ligand molecules that fit these receptors following the logic of the key–lock model [14,41,42,43]. A radioactive tracer synthesized by Pfizer (“CP55, 940”) has enabled researchers to map the locations of cannabinoid receptors in the brain. These receptors have been identified in the central nervous system (CNS) and in high concentrations in regions responsible for mental and physiological processes, such as the hippocampus (memory), cerebral cortex (cognition), cerebellum (motor coordination), basal ganglia (movement), hypothalamus (appetite), and amygdala (emotions) [35,44]. There are fewer cannabinoid receptors, more precisely, CB1Rs, identified in the brainstem, the region that controls breathing and the heartbeat, which may explain the fact that there have never been reports of overdose deaths from *Cannabis* use, regardless of age, its clinical purpose, or the route of administration [45,46]. Anxiety, paranoia, and coughing fits were the most prevalent adverse reactions to *Cannabis* intoxication, whereas cold sweats, other hallucinations (non-auditory/visual), and weight gain were the three least common related reactions. Chest discomfort, vomiting, and body humming were also experienced in reaction to *Cannabis* [47].

In addition to neurons, CB1R is expressed, albeit to a much lesser extent, in astrocytes, oligodendrocytes, and microglia, which have been shown to mediate synaptic transmission [46,48,49]. Previous studies have suggested that CB1Rs are highly expressed at presynaptic terminals and modulate retrograde endocannabinoid signaling [50]. However, the existence of CB1Rs at postsynaptic sites has not been excluded, such as in functional studies demonstrating the autoinhibition of neocortical neurons by endocannabinoids [51]. Studies involving the mapping of the rat brain suggest that the preferred location of CBR1 is in axons and nerve terminals and that its actions are related to the modulation of the release of neurotransmitters such as norepinephrine, dopamine, acetylcholine, glutamate, 5-hydroxytryptamine, γ-aminobutyric acid (GABA), and d-aspartate [1,52,53].

CB1R is abundantly expressed in the peripheral nervous system as well as in other regions of the body [54,55]. In the PNS, CB1R is highly expressed in the sympathetic nerve terminals. Furthermore, CB1Rs are observed in the trigeminal ganglion, dorsal root ganglion, and dermal nerve endings of primary sensory neurons, where they regulate the nociception of afferent nerve fibers. In the gastrointestinal tract (GIT), CB1R is expressed both in the enteric nervous system and in non-neuronal cells such as the intestinal mucosa, including enteroendocrine cells, immune cells, and enterocytes. CB1Rs modulate GIT mobility [56], the secretion of gastric acids [57], fluids [58], neurotransmitters [59], and hormones [60] as well as the permeability of the intestinal epithelium [61]. CB1Rs present in the CNS display roles in the modulation of appetite in the hypothalamus and regulate energy balance and food intake in the GIT. Interestingly, hepatic CB1Rs may also participate in the regulation of energy balance and metabolism [46,62,63].

Normally, CB1R expression in the liver is very low; however, under pathological conditions, CB1R expression in various liver cell types is remarkably increased, where CB1Rs actively contribute to hepatic insulin resistance, fibrosis, and lipogenesis. Likewise, CB1R is upregulated in the cardiovascular system under pathological conditions, promoting disease progression and cardiac dysfunction [55,64,65,66].

Oxidative stress, inflammation, and fibrosis have been observed following CB1R activation in cardiomyocytes, vascular endothelial cells, and smooth muscle cells. In addition to the mentioned tissues, CB1R expression has also been reported in adipose tissue, skeletal muscle, bone, skin, eyes, the reproductive system, and various cancer cell types. The skeletal muscle and myocardial CB1Rs are predominantly located in the mitochondria (mtCB1R). The activation of mtCB1 receptors may participate in the mitochondrial regulation of oxidative activity, probably through relevant enzymes involved in the metabolism of pyruvate, the main substrate for tricarboxylic acid activity [23,67,68,69].

The type 2 cannabinoid receptor (CB2R) was cloned in 1993 from human promyelocytic leukemia cells of the HL-60 lineage [15], and it was further identified in mice, rats, zebrafish, and dogs [70,71,72,73]. It has an amino acid sequence with approximately 44% homology to CB1R amino acid residues. CB2R is mainly found in cells of the immune system, where its expression levels have been found to be higher than those of CB1R [24,46,74].

CB2Rs modulate immune cells and contribute to the analgesic and/or antinociceptive effects of cannabinoids. CB2Rs have been identified in the CNS. However, some studies have shown their presence on the surfaces of microglia and neurons located in the cerebellum, brainstem, thalamus, striatum, cortex, amygdala, and hippocampus [46,75].

**Figure 2:**
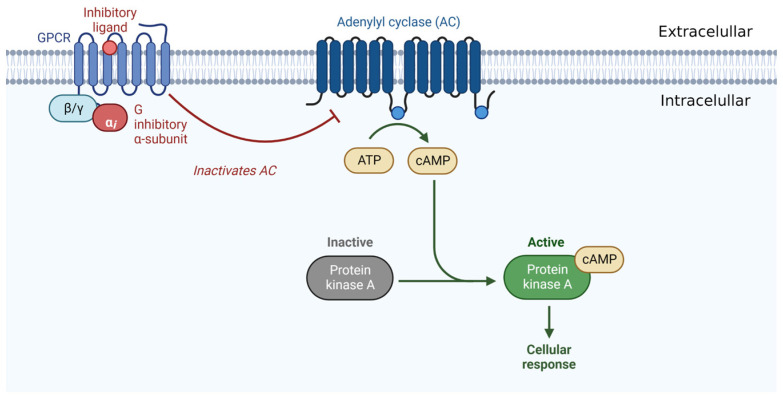
Illustration of a receptor belonging to the large family of GPCRs.

Both CB1Rs and CB2Rs belong to a large family of G-protein-coupled receptors (GPCRs). They belong to a family of membrane proteins that have an amino-terminal extracellular domain, seven conserved transmembrane helices with a characteristic sequence of 20 to 27 amino acid residues with high hydrophobicity, three extracellular and three intracellular loops, and an intracellular carboxylic acid domain terminal [40,76] (Figure 2).

**Figure 3:**
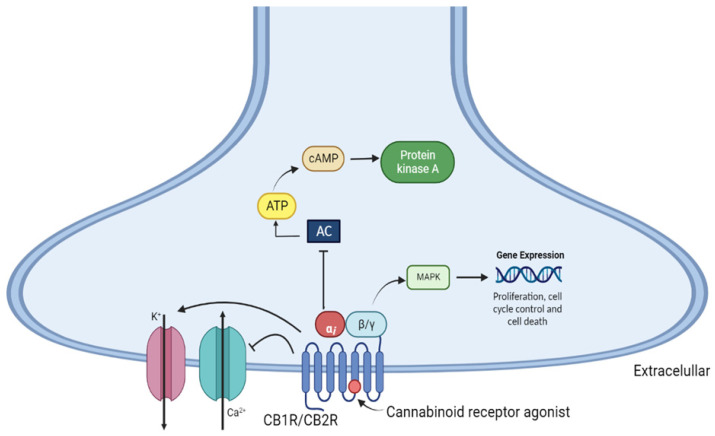
Mechanism of intracellular signaling via activation of CB1 and CB2 cannabinoid receptors. AC—adenylate cyclase, cAMP—cyclic adenosine monophosphate; ATP—adenosine triphosphate; MAPK—mitogen-activated protein kinase; PKA—protein kinase A.

The activation of both cannabinoid receptors promotes adenylate cyclase inhibition in various cell types through coupling with the Gi/o protein. This leads to decreases in the levels of adenosine 3′,5′-monophosphate (cAMP) and protein kinase A activity (PKA), which may be associated with nociceptive neuron sensitization, and proteins that might be related to increased intracellular calcium, inositol triphosphate, and diacylglycerols, which are ultimately involved in the modulation of neurotransmitter release [77,78]. CB1R stimulation leads to the activation of the mitogen-activated protein kinase (MAPK) signaling pathway, including extracellular signal-regulated kinase 1/2 (ERK1/2), c-Jun N-terminal kinase (JNK), and p38, which are involved in the regulation of cell proliferation, cell cycle control, and cell death [46,79] (Figure 3).

## 3. Endocannabinoids: Synthesis, Release, and Metabolism

With the discovery of cannabinoid receptors, there has been interest in finding endogenous ligands that are responsible for their modulation. An evaluation of purified porcine brain fractions led to the identification of a new compound that binds to CB1R. Arachidonylethanolamide, an arachidonic acid derivative in the porcine brain, was characterized and named anandamide (AEA), a word derived from the Sanskrit word ananda, which means extreme happiness [18,77,80,81,82].

**Figure 4:**
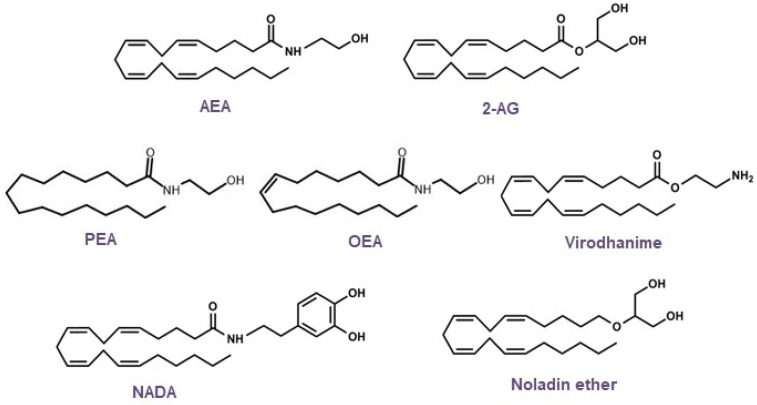
Chemical structures of the main endocannabinoids.

Based on the structural elucidation of AEA, other endogenous lipid molecules were identified (Figure 4) and are generally called *N*-acylethanolamines (NAEs), such as 2-arachidonoylglycerol (2-AG), *N*-oleoylethanolamine (OEA), 2-arachidonyl glyceryl ether (noladin, 2-AGE), virodhamine, *N*-arachidonoyldopamine (NADA), and *N*-palmitoylethanolamine (PEA). AEA and 2-AG are the most studied endogenous ligands; however, research on endocannabinoids has since been conducted, and additional receptors, along with their lipid mediators and signaling pathways, have been revealed [81,83,84,85,86,87,88,89,90,91,92,93].

Endocannabinoids, unlike classical neurotransmitters, are considered atypical messengers because of the modulation of information from postsynaptic terminals to presynaptic terminals, which is known as the retrograde signaling mechanism. Endogenous ligands are synthesized on demand or by activity dependent on the cleavage of the phospholipid membrane and are released immediately after their biosynthesis to act as pro-homeostatic factors through interactions with specific receptors [77,94,95].

The synthesis and degradation of endogenous cannabinoid receptor ligands involve different enzymatic reactions. AEA biosynthesis occurs through its release from membrane phospholipids and can follow the Ca^2+^-dependent *N*-acyltransferase (NAT) or Ca^2+^-independent *N*-acyltransferase (iNAT) pathways. Therefore, *N*-arachidonoyl-phosphatidylethanolamine (NArPE) is formed, and by the action of *N*-Acyl-phosphatidylethanolamine-specific phospholipase D (NAPE-PLD), NArPE is converted to *N*-arachidonoylethanolamine (AEA) [55,96,97].

Another endogenous ligand, 2-AG, is formed via a two-step mechanism. Initially, 1,2-diacylglycerol (DAG) is synthesized after the cleavage of a membrane phospholipid by the phospholipase C (PLC) enzyme. DAG is subsequently esterified by the enzyme diacylglycerol lipase (DAGL), creating 2-AG [98,99].

**Figure 5:**
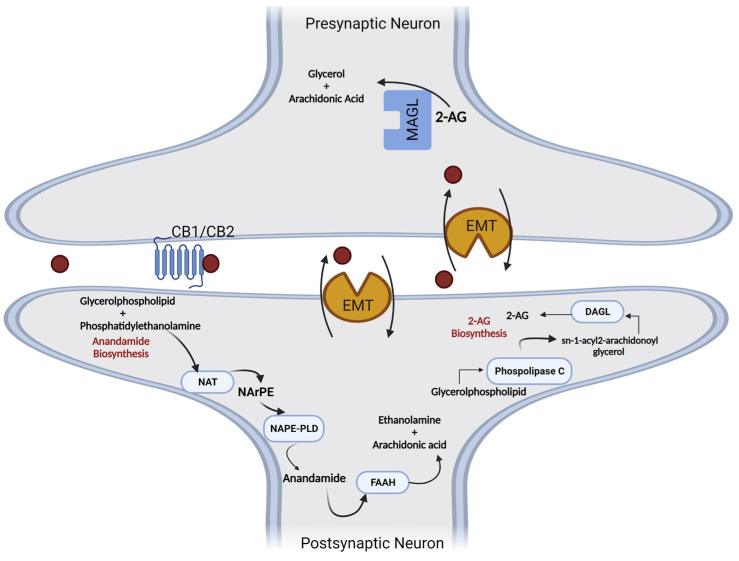
Metabolic pathways involved in the synthesis and degradation of anandamide (5) and 2-arachidonyl glycerol (6). AA—arachidonic acid; 2-AG-2—arachidonylglycerol; DAGL—diacylglycerol lipase; EMT—membrane transporters; FAAH—fatty acid amide hydrolase; MAGL—monoacylglycerol lipase; NAPE-PLD—*N*-arachidonylphosphatidylethanolamine phospholipase D; NArPE—*N*-acylphosphatidylethanolamine; NAT—*N*-acyltransferase; PLC—phospholipase C.

Endogenous cannabinoids become inactive through a cellular reuptake mechanism involving membrane transporters (EMT), followed by intracellular degradation through the action of hydrolytic enzymes. Anandamide is mainly metabolized by the fatty acid amide hydrolase enzyme (FAAH), and 2-AG is a substrate of monoacylglycerol lipase (MAGL), which produces arachidonic acid (AA) and glycerol [100,101] (Figure 5).

Furthermore, AEA and 2-AG may be susceptible to oxidative mechanisms catalyzed by cyclooxygenases (COXs), lipoxygenases (LOXs), and enzymes involved in the oxidation of arachidonic acid (AA), which is biotransformed into prostaglandins (PG), eicosanoids, and hydroxy-peroxy-anandamide, among other compounds derived from this metabolic reaction [55,77].

The endocannabinoid deficiency theory is based on the concept that many brain disorders are associated with a deficiency of neurotransmitters, such as acetylcholine in Alzheimer’s disease (AD), dopamine in Parkinsonian syndromes, and serotonin and norepinephrine in depression, and a comparable deficiency in endocannabinoid levels might similarly manifest in certain disorders that exhibit predictable clinical features as sequelae of this deficiency [102,103,104].

In 2004, Professor Dr. Ethan Russo and his coworkers proposed clinical endocannabinoid deficiency syndrome (CDS), suggesting that an endocannabinoid depletion (hypofunctional eCB) could cause many diseases, such as migraine, a highly complex disease that involves signaling between different areas of the brain and various neurochemical transmitters. The exact cause of migraine is not fully understood, although genetic predisposition is considered a primary contributor to its genesis and modulation [102,104]. The possible relationship between migraine and the endocannabinoid system has been highlighted by several studies [105,106].

Fibromyalgia is also related to deficiencies in the endocannabinoid system and is characterized by acute and chronic widespread musculoskeletal pain throughout the body. This pain is more often accompanied by other conditions such as insomnia, migraine, mood swings, memory problems, irritable bowel syndrome (IBS), and chronic fatigue. The presence of characteristic painful nodules, known as trigger points, is notable and particularly prevalent in the shoulders and neck. Similar to migraine, fibromyalgia is associated with hyperalgesia, a lowered pain threshold associated with central endocannabinoid hypofunction in the spinal cord. According to Russo et al., the approved drugs for fibromyalgia, duloxetine, milnacipran (serotonin and adrenergic inhibitors, respectively), and pregabalin (an anticonvulsant used to treat neuropathic pain) showed little efficacy in treating fibromyalgia compared to *Cannabis* [106,107,108].

IBS, also known as spastic colon, is a functional disorder characterized by GIT pain, spasm, discomfort, and altered bowel movements, predominantly diarrhea. GIT propulsion, secretion, and inflammation in the gut are modulated by the endocannabinoid system, providing a rationale for the inclusion of cannabinoids in IBS treatment [109]. Studies have shown that increased capsaicin receptor TRPV1 expressing sensory fibers may contribute to visceral hypersensitivity and pain in IBS and provide a new therapeutic target. Cannabidiol could be used for therapeutic interventions because of its effect on vanilloid VR1 receptors; it also enhances anandamide signaling. Its analogs have been shown to be potent inhibitors of anandamide cellular uptake [110,111,112,113,114,115].

Neurodegenerative disorders may lead to the development of Parkinson’s disease (PD) and AD. Normally, they are characterized by cognitive impairment and other neurological defects. Currently, the endocannabinoid system is studied as a drug target in PD and AD because of the overexpression of endocannabinoid system receptors, which exert neuroprotection against PD and reduce neuroinflammation in AD. Increased levels of AEA were found in the cerebrospinal fluid of untreated patients with PD, which was suggested to be a possible compensatory mechanism. Cognitive deficits in AD patients correlate with cerebral disturbances in sensitive brain areas, largely in the frontal cortex and hippocampal regions, which are rich in CB1Rs. Δ9-THC and CBD showed neuroprotection in PD and AD animal models; however, they triggered toxic effects in patients when administered directly. Studies are necessary to determine the therapeutic efficacy of targeting the endocannabinoid system in neurodegenerative diseases [101,116,117,118].

In some cases, eCB might be hyperfunctional, promoting cognitive deficits that may be noticeable in fragile X syndrome (FXS), Down syndrome, and Williams–Beuren syndrome (WBS). In addition to the genetic causes of these syndromes, it is believed that eCB is overactivated. In an animal model of FXS, knockout mice with fragile X mental retardation protein (Fmr1) recapitulate the main features of the disease. BlockingCB1R and CB2R in male Fmr1 knockout mice normalized the cognitive impairment and anxiolytic-like behavior, respectively [119]. In a preclinical model of Down syndrome, the segmental trisomic Ts65Dn mouse model showed that CB1R expression was enhanced, and its function increased in hippocampal excitatory terminals. The knockdown and inhibition of CB1R repaired memory deficits in male Ts65Dn mice [120]. To evaluate a model mimicking WBS, mice with the same genetic deletion found in patients with WBS were used. Male mice showed hypersocial behaviors, memory deficits, enlarged hearts, and differences in the function of CB1R. These mutant mice received JZL184, an MAGL inhibitor, which improved their social and memory symptoms and cardiovascular function [121]. These studies show that the modulation of eCB hyperactivity is a promising therapeutic approach for cognitive deficits associated with genetic syndromes.

## 4. Molecules That Modulate the Endocannabinoid System

**Figure 6:**
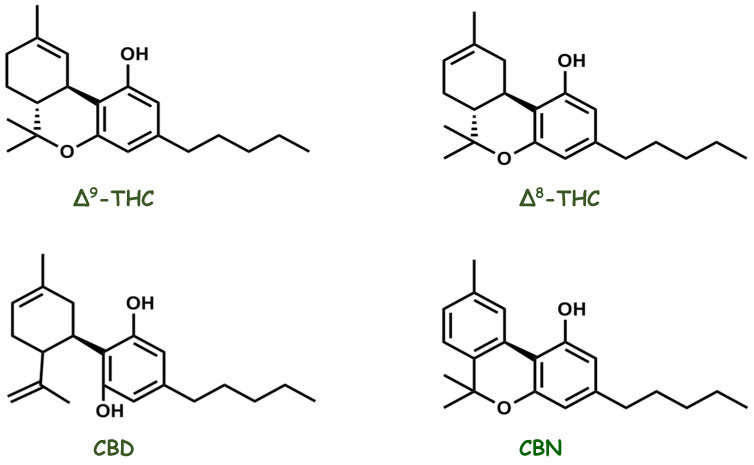
Chemical structures of the main pharmacologically active cannabinoid compounds isolated from *Cannabis sativa*.

*Cannabis*, an herbal medicine, is a complex mixture of several compounds, including cannabinoid phenols, non-cannabinoid phenols (simple phenols, spiro-indans, dihydrophenanthrenes, and dihydrostilbenes), flavonoids, terpenoids, alcohols, aldehydes, n-alkanes, wax esters, steroids, and alkaloids. In 1899, Wood isolated cannabinol (CBN), the first compound purified from the plant. Currently, more than 500 different substances have been isolated and reported from *Cannabis* plants belonging to different classes, among which the class of cannabinoid compounds is the most representative because it has more than 120 identified compounds, such as delta-eight and delta-nine tetrahydrocannabinol (Δ8-THC and Δ9-THC), CBD, and CBN (Figure 6). Diverse classes of secondary metabolites from different parts of the plant have been identified, with a wide range of applications (nutraceuticals, cosmetics, aromatherapy, and pharmacotherapy) that are beneficial to humans. However, in the past, studies were focused on the two most abundant phytocannabinoids, THC and CBD, thus resulting in greater knowledge about their pharmacological activities and increasing interest in the numerous possibilities of the medicinal actions of the plant [109,122,123,124,125,126,127,128]. CBD has been gaining prominence in pharmacological research since the 1970s. Epidiolex^®^, a purified oral CBD medicine, is currently approved by the U.S. Food and Drug Administration for the treatment of intractable childhood-onset seizures [129,130].

However, the biochemical basis of the pharmacological activity of cannabinoids has remained an enigma for many years. The highly lipophilic molecular structure of cannabinoids suggests that they exert their effects by penetrating cell membranes and acting in the CNS. Currently, important insights into the physicochemical properties of cannabinoids are available. Novel selective ligands for cannabinoid receptors can have specific substituents that increase binding kinetics and decrease side effects [131,132,133,134,135].

## 5. Endocannabinoid System Emerging as a Pharmacotherapy Target for Chronic Pain and Mood Disorders

Pain is described as an unpleasant sensory and emotional experience associated with actual or potential tissue damage or in terms of such damage. When pain persists or recurs for longer than three months, it is defined as chronic pain and has a major impact on society. An estimated 20% of the global population suffers from chronic pain. Importantly, depression and anxiety are significantly observed in such patients [136,137,138]. Pain therapy includes both pharmacological and non-pharmacological treatment options. Antidepressants, anticonvulsants, and drugs that act on the CNS are commonly recommended for chronic pain treatment. Therapeutic agents are considered adjuvant analgesics, medications that were not primarily developed as analgesics but have pain-relieving properties, and are the first-line drugs for neuropathic pain treatment and psychiatric problems [139]. However, some patients do not show pain alleviation and seek other therapies to reverse their condition. Pain relief was already described by the Chinese in the third millennium BC due to the use of extracts of the hemp plant (*Cannabis sativa*) to cause a variety of medicinal effects. Recently, interest in the medicinal properties of *Cannabis sativa* has resurged with the emergence of the eCB, offering not only new insights into the mechanisms underlying the therapeutic actions of cannabinoid-like molecules and phytocannabinoids but also novel molecular targets for the pharmacotherapy of pain [2,140]. Studies in animal models of acute pain showed that Δ9-THC, CBD, AEA, and synthetic cannabinoids such as CP55,940 and WIN 55,212-2 had antinociceptive actions [141,142,143,144,145,146,147,148]. In a model of chronic pain, AEA and cannabinoid ligands were effective treatments [147,149,150,151,152]. The combination of endocannabinoids and synthetic cannabinoids with nonsteroidal anti-inflammatory drugs promotes synergistic antinociceptive effects and may be useful in the pharmacotherapy of pain. In addition, studies of paracetamol (acetaminophen) activity, the most widely used painkiller, suggest that its analgesic efficacy is, in part, mediated by CB1R stimulation [144,153,154,155,156,157]. Natural and synthetic cannabinoids, such as dronabinol and nabilone, have been studied in humans for chronic pain relief, and therapeutic efficacy for pain management and quality of life improvement in patients was observed. The eCB is distributed throughout the spinal and supraspinal regions, thus can effectively regulate nociceptive processing [158,159,160,161,162,163].

CB1Rs may be activated by THC, producing analgesia and adverse events (e.g., headache, numbness, cough, burning sensation, dizziness, feeling high, somnolence, and dry eyes and mouth) [164,165]. Many of the psychoactive events depend on THC concentrations and might become important disadvantages of its use as a pharmacological therapy [166]. A previous study demonstrated that CB1R activation causes memory impairment [167,168]. In addition, THC and other cannabinoids activate serotonin 2A receptors (5-HT2AR), modulating memory deficits, anxiolytic-like effects, and social interaction [169]. Previous studies have shown that CB1R and 5-HT2AR form heteromers that are expressed and functionally active in specific brain regions involved in memory impairment, such as the hippocampus and prefrontal cortex [167,170,171,172]. However, memory deficits and anxiety were abrogated in wild-type mice with the use of a 5-HT2AR antagonist or the selective disruption of the CB1R/5-HT2AR heteromers by an infusion of synthetic interference peptides without losing the antinociceptive effect [167]. Ongoing studies on the use of *Cannabis* show that it promotes pain relief and dissociated memory impairment, reducing drawbacks for the use of cannabinoids as therapeutic agents [168]. CB2R plays an important role in modulating analgesia via two pathways. The first mechanism occurs in the peripheral immune system, where CB2Rs mediate analgesia by modifying the cytokine profile and preventing adverse effects on the CNS. Secondly, CB2Rs present in glial cells and neurons contribute to pain relief [173,174]. Additionally, studies have shown that selective CB2 agonists promote antinociception [175,176].

Anxiety and panic disorders, major depressive conditions, and bipolar disorder (manic–depressive illness) are mood complications that are often serious and potentially life-threatening. More than 20% of the adult population experience mood disorders at some point in their lives [177]. Many advances have been made in mood disorder treatment over the past decades. Approximately 30% of the population does not respond to current therapies, and the search for novel pharmacological approaches continues [178,179].

The psychoactive effects of *Cannabis* include calming, anxiolytic, sleep-inducing, and euphoric effects. Some of these factors positively affect moods. However, symptoms such as paranoia, irritation, dysphoria, depression, depersonalization, and demotivation may appear in some individuals with such adverse effects [51,180]. The reactions depend on the patient’s endocannabinoid activity, the dose used (normally stimulating action at a low dose and inhibitory action at a high dose), the proportion of phytocannabinoids, and the terpenoid composition [179,181]. Evidence is increasing regarding the role of eCB in mood regulation. Clinical studies have shown altered endocannabinoid signaling in psychiatric patients [182]. Genetic polymorphisms in CB1R and CB2R are associated with major depression, bipolar disorder, and resistance to therapy, which has been observed in depressed patients who have a single-nucleotide polymorphism in CB1R [183,184,185,186]. Moreover, eCB might modulate the functions of all hypothalamic–pituitary axes via CB1R, and chronic stress seems to reduce the eCB system’s ability to suppress stress and may induce psychopathology, including depression and anxiety [187]. In this sense, it is important to remember the trajectory of the drug rimonabant, the first cannabinoid receptor blocker to be approved for metabolic syndrome treatment, obesity, and smoking. However, due to important adverse effects that became evident in patients following chronic exposure to rimonabant, Sanofi-Aventis withdrew it from the market. This drug mainly exerts its beneficial effects by blocking CB1 receptors in the periphery. However, due to its lipophilic nature, rimonabant can cross the blood–brain barrier and enter the CNS, and it is linked to the development of depression, suicidal feelings, and anxiety disorders [188,189,190]. The mood-elevating properties of cannabinoids have long been known and are considered non-toxic. Some *Cannabis* constituents or mixtures may have antidepressant and/or anxiolytic effects. Many patients who are nonresponsive to the usual pharmacological treatments for depression may benefit from medicinal *Cannabis* use. Cannabinoids may have therapeutic potential for both depression and bipolar disorder. This is related to some patients adding *Cannabis* to ongoing treatment since this association might improve the efficacy of such medication and/or reduce its side effects. *Cannabis* may be a mood stabilizer in bipolar disorder and an adjuvant to lithium treatment. Patients experiencing a mood disorder may not be objective in assessing their condition and cannot decide on their own to modify the treatment. Thus, professional care and control are essential [177,179,191,192,193,194].

## 6. Harmonization of the Endocannabinoid System through Integrative and Complementary Health Practices (ICHP)

Clinical interventions are characterized as integrative and complementary health practices (ICHP), also known as “complementary and alternative medicine (CAM)”, which include various medical and health systems, practices, and products that are not currently part of conventional therapies. CAM is classified into three broad groups: “natural products” (dietary and herbal supplements), “mind and body medicine” (meditation, yoga, and acupuncture), and “body-based practices” (massage and spinal manipulation) [195,196].

A rodent study conducted by Chen et al. showed that electroacupuncture promoted antinociceptive activity in animals and increased AEA levels in skin tissue. It was also found that the antinociceptive effects were attenuated when using AM630, a CB2R antagonist, but not when using the CB1R antagonist AM25 [197]. Furthermore, AEA increased the expression of CB2R in the skin [198,199]. CB2R activation in the skin likely stimulates the release of β-endorphin, which then acts on peripheral μ-opioid receptors to inhibit nociception [51]. Furthermore, electroacupuncture conferred neuroprotection against cerebral ischemia by stimulating the mobilization of endocannabinoids in the brain and activating CB1R [200,201].

Sadhasivam et al. suggested that endocannabinoids may serve as biomarkers after a meditation session. Depression and anxiety scores significantly decreased and happiness and positive well-being scores were enhanced after four days of an Isha Yoga Bhava Spandana program. Additionally, one day before and one day after, blood samples were collected voluntarily for the evaluation of the levels of AEA, 2-AG, 1-AG, docosatetraenoylethanolamide (DEA), oleoylethanolamide (OLA), and brain-derived neurotrophic factor (BDNF). Analyses suggest that major endocannabinoids, including AEA, 2-AG, 1-AG, DEA, and BDNF, increased after meditation in >70% of patients, suggesting an important role for these biomarkers in the mechanism underlying meditation [202]. Studies have indicated that there is a correlation between acupuncture and eCB through the biological effects shared by both, including analgesia, neuroprotection, and cardiovascular regulation. A better understanding of these intrinsic links between acupuncture and CES may allow for the development of new techniques that combine acupuncture with therapeutic agents that target the endocannabinoid lysis signal [203,204,205,206].

Another study found that massage and osteopathic manipulation of asymptomatic participants increased serum AEA levels by 168% compared to pretreatment levels; there were no changes in 2-AG levels. Participants who underwent sham manipulation (control) showed no changes [207]. An integrative approach combining acupuncture, massage, yoga, mind–body approaches, and medical *Cannabis* might be quite effective. As an example, we have a patient with chronic neuropathic pain showing improvement in the clinical picture when treated this way [208]. Accordingly, a complex individualized approach is needed, highlighting patient guidance and engagement in integrative modalities and the medicinal use of *Cannabis*.

## 7. Research Perspectives and Trends in the Endocannabinoid System

Since the beginning of scientific research with cannabinoids, with a special emphasis on the isolation and identification of phytocannabinoids such as THC, scientists have continued to improve, day after day, the knowledge of the pharmacology, biochemistry, and clinical effects of *Cannabis*. For years, the physiological effects of its consumption have been well known, particularly in the state of euphoria. However, what occurs inside our bodies at the molecular level, especially in the brain, to alter consciousness is still unknown. In 1973, US researchers identified receptors in the brain that are linked to opiates. Some scientists expected the discovery of receptors for marijuana to occur rapidly. However, it was not as easy or as fast as they wanted. Research by Allyn Howlett and William Devane identified that cannabinoid receptors were more abundant in the brain than any other GPCR [206,209,210,211].

CB1R and CB2R, as part of the endocannabinoid system, play critical roles in numerous physiological conditions and human diseases. Therefore, considerable efforts have been made to develop ligands for CB1R and CB2R, resulting in hundreds of phytocannabinoids and synthetics that have shown varied affinities for the treatment of various diseases [17]. However, only a few of these ligands have been used clinically. Currently, more detailed structural information for cannabinoid receptors has been revealed by cryoelectronic microscopy, which has accelerated the discovery of structure-based substances [209]. At the same time, new peptide-like cannabinoids of animal origin arrived on the scene, with potential therapeutic effects in vivo on cannabinoid receptors [212,213].

From the point of view of natural products, it is expected that new cannabinoids will be discovered and predicted as prototypes for promising drugs from different sources and natural species, such as animal venoms, which constitute a true pharmacopeia of toxins modulating diverse targets, including ion channels and GPCRs such as CB1R and CB2R, with significant affinity and selectivity [214]. Therefore, it is believed that discovering new cannabinoids by studying the biodiversity of species that live on Earth is a territory that has yet to be explored.

## 8. Conclusions

The roles of cannabinoid receptors and their agonists in multiple conditions have been addressed in this review. Since research with derivatives of *Cannabis* has started and the biological functions of isolated compounds in experimental and human diseases have shown promising outcomes, it is evident that selective ligands of specific Cannabis receptors could induce beneficial outcomes, depending on the clinical condition. More research on the biological function of each *Cannabis* derivative should be encouraged.

## Data Availability

Not applicable.
